# Preferential Antibody and Drug Conjugate Targeting of the ADAM10 Metalloprotease in Tumours

**DOI:** 10.3390/cancers14133171

**Published:** 2022-06-28

**Authors:** Hengkang Yan, Mary E. Vail, Linda Hii, Nancy Guo, Paul J. McMurrick, Karen Oliva, Simon Wilkins, Nayanendu Saha, Dimitar B. Nikolov, Fook-Thean Lee, Andrew M. Scott, Peter W. Janes

**Affiliations:** 1 Tumour Targeting Program, Olivia Newton-John Cancer Research Institute, Heidelberg, VIC 3084, Australia; hengkang.yan@onjcri.org.au (H.Y.); mary.vail@onjcri.org.au (M.E.V.); nancy.guo@onjcri.org.au (N.G.); fsejlee@bigpond.net.au (F.-T.L.); 2 School of Cancer Medicine, La Trobe University, Heidelberg, VIC 3084, Australia; 3 Monash Biomedicine Discovery Institute, Department of Biochemistry and Molecular Biology, Monash University, Clayton, VIC 3800, Australia; linda.hii@monash.edu; 4 Cabrini Monash University Department of Surgery, Cabrini Hospital, Malvern, VIC 3144, Australia; pjm@colorectal.com.au (P.J.M.); kaz@bigpond.net.au (K.O.); simonwilkins@cabrini.com.au (S.W.); 5 Department of Epidemiology and Preventive Medicine, Monash University, Melbourne, VIC 3004, Australia; 6 Structural Biology Program, Memorial Sloan-Kettering Cancer Centre, New York, NY 10065, USA; sahan@mskcc.org (N.S.); nikolovd@mskcc.org (D.B.N.)

**Keywords:** ADAM metalloprotease, therapeutic antibody, antibody–drug conjugate, colon cancer, brain cancer

## Abstract

**Simple Summary:**

ADAM10 is a cell surface protein that releases other proteins from cells, thereby controlling a range of functions during normal development. Its activity is normally tightly regulated, but it can become deregulated in cancer calls. We previously showed that an active form of ADAM10 is elevated in tumours in both mice and humans, and that we could detect this active form using an antibody which we developed that binds to a specific region of ADAM10, which is apparently hidden in the inactive form. We now provide insight explaining the specificity of our antibody (8C7) for active ADAM10, and show that it preferentially targets tumours when injected into mice. We thus conjugated cytotoxic drugs to 8C7 in order to preferentially bind and kill tumour cells that contain activated ADAM10. Our experiments show that our ‘8C7 antibody–drug conjugates’ specifically kill cells expressing 8C7-reactive ADAM10, and can inhibit the growth of tumours in mice, without significant side effects, suggesting their potential as a novel approach for targeted cancer therapy.

**Abstract:**

ADAM10 is a transmembrane metalloprotease that sheds a variety of cell surface proteins, including receptors and ligands that regulate a range of developmental processes which re-emerge during tumour development. While ADAM10 is ubiquitously expressed, its activity is normally tightly regulated, but becomes deregulated in tumours. We previously reported the generation of a monoclonal antibody, 8C7, which preferentially recognises an active form of ADAM10 in human and mouse tumours. We now report our investigation of the mechanism of this specificity, and the preferential targeting of 8C7 to human tumour cell xenografts in mice. We also report the development of novel 8C7 antibody–drug conjugates that preferentially kill cells displaying the 8C7 epitope, and that can inhibit tumour growth in mice. This study provides the first demonstration that antibody–drug conjugates targeting an active conformer of ADAM10, a widely expressed transmembrane metalloprotease, enable tumour-selective targeting and inhibition.

## 1. Introduction

A disintegrin and metalloprotease (ADAM) 10 is a transmembrane protein with extracellular metalloprotease (MP), disintegrin (D) and cysteine-rich (C) domains. It is an active protease and it sheds a range of proteins from the cell surface, including growth factors, cytokines and their receptors, cell adhesion molecules and cell guidance receptors, thereby regulating a variety of signalling processes important in both normal and oncogenic development [1,2,3]. Prominent substrates include the Notch receptor family, where ADAM10 is the main α-secretase, mediating ligand-dependent Notch extracellular domain shedding, triggering subsequent γ-secretase-mediated release of the Notch intracellular domain (NICD) [4,5]. The released NICD directly controls transcriptional programs regulating cell fate, proliferation and survival, and contributes to angiogenesis and stem cell maintenance [6]. ADAM10 expression and ADAM10-dependent signalling is associated with poor prognosis in a range of cancer types, including gastrointestinal, breast, prostate, ovarian and brain cancers [1,7]

Given the wide variety of reported substrates, it is not surprising that the sheddase activity of ADAM10 is tightly controlled. This occurs via multiple mechanisms, including regulation of its transport to the cell surface by tetraspanins [8]; multimerization and interactions with other regulatory proteins, such as tissue inhibitors of metalloproteases (TIMPs) [9]; and substrate recognition via a pocket within the non-catalytic C domain, which facilitates positioning of the substrate to enable its access to the catalytic site [10,11]. Intramolecular interactions also control access of substrate to the protease catalytic site. The Pro domain of ADAM10 is thought to act as a chaperone, and to block the catalytic site, and it is removed by furin to produce the mature (processed) form of ADAM10, although this is not necessarily required for activity [12]. In addition, X-ray crystallography of the mature ADAM10 extracellular domain indicates a bent structure where the N-terminal MP domain interacts with the membrane proximal C domain, such that the catalytic site is partially occluded [10,13]. This may also obstruct substrate access to the substrate-binding pocket in the C domain [11]. Thus, it is likely that a conformational change within the ADAM10 extracellular domain (ECD) is required to expose the active and substrate binding sites, in order to facilitate substrate proteolysis. Indeed, studies involving the closely related ADAM17 indicate conformational changes in the ECD, regulated by changes in intramolecular disulphide bonding between cysteine motifs conserved also in ADAM10, which are thought to regulate accessibility of substrate to the catalytic site [14,15,16]. This ‘disulphide switch’ is catalysed by protein disulfide isomerases (PDIs), the activity of which is dependent on oxidative conditions [17]. Accordingly, ADAM proteinase activity of ADAMs is enhanced by oxidative conditions, such as those that occur during inflammation processes, and in the tumour microenvironment [18].

We previously raised a monoclonal antibody (mAb) against the ADAM10 D and C domain region which is specific for ADAM10, and we showed that its binding correlates with ADAM10 activity [13,19,20]. Structural studies of mAb 8C7 in complex with the isolated ADAM10 D and C domain region bound to 8C7 defined the binding epitope within the C domain. Mutation of a key CxxC disulphide-switch consensus motif adjacent to the 8C7 epitope ablated its binding, consistent with conformation-dependence [20]. 8C7 binding also correlated with enhanced ADAM10 activity against peptide substrates in vitro, indicating its recognition of a form of ADAM10 in which the catalytic site is accessible, or open [13,20]. Importantly, 8C7 preferentially bound ADAM10 from tumours versus that from normal tissues, indicating enhanced ADAM10 activity in tumours, and therefore the potential of 8C7 to be developed as a tumour-targeting agent [20]. We thus set out to further define the selectivity of 8C7 for active ADAM10, and to investigate the potential of novel 8C7 antibody–drug conjugates (ADCs) for the selective delivery of cytotoxic drugs to tumours.

## 2. Materials and Methods

### 2.1. Cell Culture and Reagents

Authenticated human LIM1215 colon and U251MG glioma cell lines from ATCC were maintained in RPMI 1640/10% FCS, while HEK293 cells were maintained in DMEM 10% FCS. ADAM10 monoclonal antibodies 8C7 and 4A11 have been previously described [19,20]. Commercially available antibodies were obtained: Phospho-Histone H2AX (Ser139) (clone 20E3) and Notch1 (clone D1E11) from Cell Signalling Technologies, Danvers, MA, USA), CD31 (clone MEC13.3, BD Pharmingen, San Diego, CA, USA) and pan-actin (ACTN05 (C4), Thermofisher, Waltham, MA, USA).

### 2.2. PCR/Cloning

The hAdam10 D + C domains (AA454–673) were amplified by PCR and cloned into the pDisplay vector (Thermofisher, Waltham, MA, USA) using BglII and SalI restriction enzyme sites (retaining the N terminal HA tag and Myc at the membrane). The construct was transfected into HEK293 cells (Fugene, Promega), and a stable line was generated through antibiotic (neomycin) selection.

For quantitative PCR, cDNA generated from RNA extracts (QIAGEN RNeasy) of snap-frozen tumor samples was analysed using an iTaq Universal SYBR Green kit (Bio-Rad Laboratories, Hercules, CA, USA) and a RotorGene 3000 cycler (Corbett Research). Primers specific for human ADAM10 were used to determine expression by comparative CT (ΔΔCT), relative to the averaged expression of three housekeeping genes (GAPDH, Beta 2 microglobulin, and Eukaryotic translation initiation factor 4E).

### 2.3. ADC Generation and Testing In Vitro

ADCs were generated by Levena Biopharma (San Diego, CA, USA) via maleimide addition to the reduced interchain cysteine thiols of antibody 8C7 and control IgG, as described previously [21] (see also Supplementary material). Monomethyl auristatin E (MMAE) was conjugated using the lysosomally cleavable linker MC-vc-PAB (maleimidocaproyl-valine-citrulline-p-aminobenzoyloxycarbonyl), achieving a drug:antibody ratio (DAR) of 3.7–4.2. The pyrrolobenzodiazepine dimer SG3199 (PBD) was conjugated with the cleavable linker MA-PEG8-VA-PAB, achieving a DAR of 3.1–3.2. The topoisomerase inhibitor DXd (DX8951) was attached with the linker MC-GGFG, for a DAR of 6.9–7.5. 

DARs were determined by hydrophobic interaction chromatography (HIC-HPLC), via relative percent peak integration of the ADC at 280 nm absorbance. The integrity and low (<2%) aggregation of the ADC were verified by size exclusion chromatography (SE-HPLC). HIC-HPLC - Column: Tosoh TSKgel Butyl-NPR, 4.6 mm I.D. × 3.5 cm, 2.5 μm particle size; Buffer A: 20 mM sodium phosphate, 1.5 M ammonium sulfate, pH 7.0; Buffer B: 20 mM sodium phosphate, 25% *v*/*v* isopropanol, pH 7.0. SEC-HPLC: Column: Tosoh TSKgel G3000SW-XL, 7.8 mm × 30 cm, 5 mm; Buffer A: 150 mM sodium phosphate, 300 mM sodium chloride, pH 7.0; Agilent 1100 HPLC, Run time 20 min. 

Effects of ADC treatment (at the indicated concentration ranges) on cell viability were measured using an MTS assay (CellTiter 96, Promega) after 3–4 days.

### 2.4. Animal Studies

Immuno-deficient athymic mice (BALB/c nude) or NSG mice (Nod/SCID/gamma) (5–6 weeks old) were obtained from the Animal Resources Centre (Canning Vale, Western Australia, Australia). All animals were handled in strict accordance with good animal practice, as defined by the National Health and Medical Research Council (Australia) Code of Practice for the Care and Use of Animals for Experimental Purposes, and approved by the Austin Hospital Animal Ethics Committee. Patient-derived xenografts were generated by subcutaneous insertion of 2–3 mm square samples of fresh colorectal tumours into the flank of NSG mice, and serial passage in NSG mice.

### 2.5. Biodistribution Studies

8C7 was coupled to ^111^In with the bifunctional metal ion chelator CHX-A diethylene-triamine-penta-acetic acid (DTPA), and to ^125^I by direct coupling using chloramine-T, as described previously [22], following which binding to immobilised antigen was confirmed. For the biodistribution experiments, BALB/c nude mice were injected subcutaneously on the flank with 10^7^ LIM1215 cells in 30% growth factor-reduced Matrigel (BD), and after 30 days (at a tumour size of approximately 250 mm^3^), the mice received a tail vein injection of ^111^In-8C7 (5 ug/8 µCi) and ^125^I (5 ug/7.05 µCi). Five mice/time point were sacrificed after 2 h, 2 d and 5 d, and tissue, blood and tumour samples were taken and weighed. Sample radioactivity was determined using a gamma counter, calculated as a fraction (%) of injected dose per gram of tissue, and graphed for each time point. For imaging, only ^111^In-8C7 [47.4 µg/75.9 µCi] was injected, and gamma camera/MRI scans (Mediso, Budapest, Hungary) were taken of anaesthetised mice.

### 2.6. Therapeutic Studies

Mice were injected subcutaneously on the flank with 10^7^ LIM1215 colon cancer cells in 30% growth factor-reduced BD Matrigel (BALB/c nude mice), or 5 × 10^6^ U251MG glioma cells in PBS (NSG mice). When tumours reached 100–200 mm^3^, randomised groups of *n* ≥ 5 mice were treated twice weekly with I.P. injection of 8C7-PBD control IgG-PBD (0.3–0.5 mg/kg), naked 8C7 (10 mg/kg) or vehicle control (PBS). Tumour volumes were determined by caliper measurement (volume = length × width^2^/2) and mouse weights were measured over the treatment course. Mice were humanely euthanized at or before the ethical endpoint, and tumours were recovered for analysis. Statistical significance was determined using the student’s T test. Results are representative of at least 2 replicate experiments.

### 2.7. Flow Cytometry, Histology and Western Blot Analysis

Flow cytometry was performed on live cells using a BD LSR II flow cytometer and a BD FACS Canto II (BD Biosciences). Tumour immunohistochemistry (IHC) and fluorescence (IF) was performed on sections (6 mm) of OCT-embedded fresh-frozen resected tumours. For IHC, sections were stained (Vector Labs ABC Kit), hematoxylin counterstained and imaged (Aperio AT2). IF staining was with Alexa-labelled secondary antibodies (Abcam) and nuclear stain (DAPI), and imaging was carried out using a Zeiss LSM 980 confocal microscope. Images were analysed with Aperio (Imagescope version 12.4.2.5010) and HALO software (version 3.1.1076.429). Western blot analysis of tumour lysates was performed as previously described [20], after equalising protein content, as determined by BSA assay (Pierce).

## 3. Results

**Figure 1:**
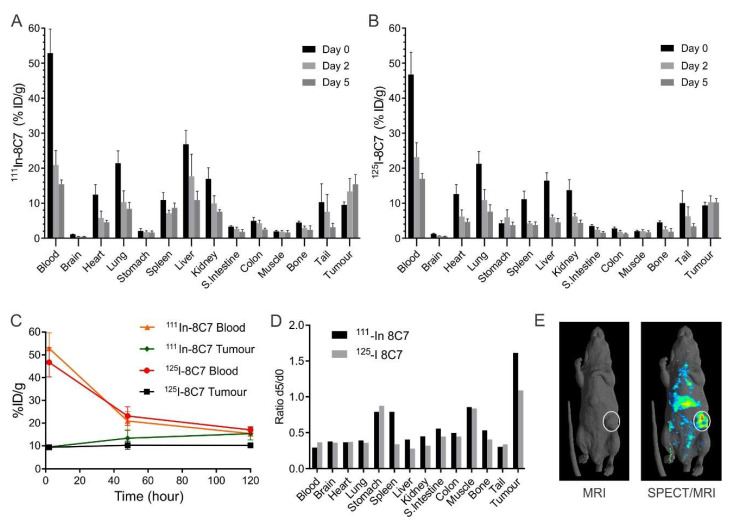
Biodistribution of radiolabelled 8C7 in mice with subcutaneous LIM1215 tumours. (**A**–**C**) Mice (*n* = 5) were injected with a mix of ^111^In- and ^125^I-labelled 8C7, and blood and tissues were recovered after 2 h, 48 and 120 h (Day 0, 2 and 5) for determination of radioactivity, expressed as mean fraction of the injected dose/gram tissue (% ID/g, ±SD). (**D**) The data in A and B showing radiolabelled 8C7 retained in tumour versus normal tissues at Day 5 relative to Day 0. (**E**) Representative images of a mouse with subcutaneous tumour (circled), injected with ^111^In-8C7 and imaged (Day 2) by surface rendered MRI and gamma camera SPECT scan.

We previously showed that our ADAM10 monoclonal antibody 8C7 is specific for ADAM10, and that it recognises both human and mouse forms, reflecting their high homology; with 95% sequence conservation in the C domain and 100% sequence conservation of residues contacting the 8C7 complementarity-determining regions (CDRs) [19]. This cross-reactivity enables assessment of the relative binding of 8C7 to ADAM10 in tumour and normal tissue in mice with human tumour xenografts. We thus evaluated the biodistribution of mAb 8C7 in mice bearing human LIM1215 colon cancer xenografts, which we have previously shown to possess high levels of active ADAM10 [20]. 8C7 was conjugated to ^111^In (Figure 1A,C) or ^125^I (Figure 1B,C) and intravenously injected, and tumour and tissue samples were collected over 6 days and analysed for radioactivity. Both conjugates showed similar distribution profiles, with gradual clearance from the blood, and clearance from most tissues consistent with blood pool clearance, with no specific tissue uptake and retention. In comparison, antibody retention in the tumour was more prolonged, indicating preferential binding to tumour cells compared to normal organs (Figure 1C,D). The enhanced tumour uptake of ^111^In-8C7 compared to ^125^I-8C7 may reflect the known rapid internalisation of 8C7 [19], as the Indium label is better retained under these conditions, while ^125^I is dehalogenated and diffuses out of the tumour cell [22]. SPECT/MRI imaging of ^111^In-8C7 2 days post injection also indicated preferential targeting of the tumour. The ^111^In-8C7 signal detected in liver and spleen was consistent with metabolism of the ^111^In-chelate in these organs, rather than with higher antibody retention (Figure 1D,E).

**Figure 2:**
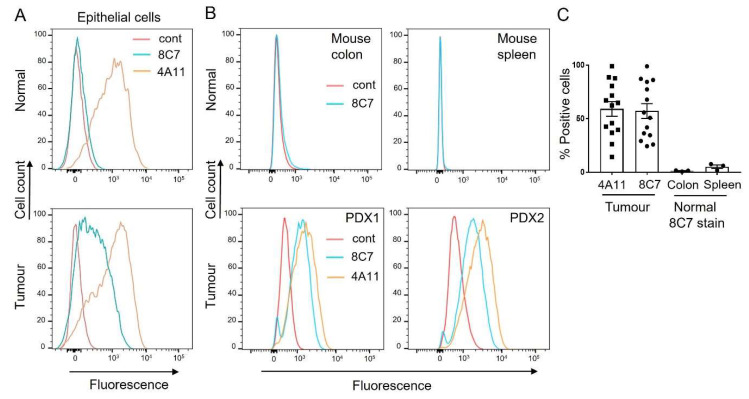
Anti-ADAM10 mAb 8C7 preferentially binds human colon tumour cells compared to normal cells. (**A**) Single cell isolates from colon tumour and matched normal patient tissue were stained with the anti-ADAM10 antibodies 8C7 and 4A11, and with antibodies against CD45 and EpCam, and analysed by flow cytometry. Histograms show normalised staining of epithelial cell populations (EpCam positive, CD45 negative). (**B**) Tumours were recovered from mice bearing two human colorectal PDX lines, and single cell isolates were stained with 8C7, 4A11 or control IgG, before analysis by flow cytometry. Normal colon and spleen were also analysed for 8C7 binding (human-specific 4A11 was not used). Histograms show overlaid binding of ADAM10 antibodies with an isotype control. (**C**) Quantitation of 8C7 binding to tumour cell isolates, from multiple passages of six distinct colorectal PDX tumour lines (*n* = 14 tumours), compared to normal tissue (% positive cells). Binding of the anti-human ADAM10 mAb 4A11 to PDX tumour cells is also shown for comparison.

We next analysed the binding of 8C7 to epithelial cells isolated from fresh human colorectal tumour and adjacent normal colon tissue. We used flow cytometry to detect the binding of fluorescent Alexa-647-labelled 8C7, or of 4A11, an anti-human ADAM10 antibody that we developed which binds to both active and inactive ADAM10 [20]. While the non-selective ADAM10 mAb 4A11 bound to both normal colon and tumour epithelial (CD45 negative, EpCam positive) cells, 8C7 preferentially bound to tumour cells, suggesting selective targeting of active ADAM10 in the tumour (Figure 2A). Quantitative PCR of ADAM10 mRNA expression confirmed comparable ADAM10 expression to that of a larger set of normal and tumour tissues from a cohort of 19 colorectal cancer patients, in which expression was independent of tumour stage (Figure S1A,B). We also generated patient-derived tumour xenograft (PDX) lines grown in immunodeficient mice, and compared 8C7 binding to tumours versus normal tissues (Figure 2B,C). 8C7 selectively bound to tumours in multiple colon PDX lines, irrespective of the initial tumour stage, compared to its low binding to normal colon, and to spleen, which was also recently reported to express ADAM10 [23]. In this case, the control antibody 4A11 did not show significant binding to normal mouse tissues (not shown), consistent with its specificity for human ADAM10 [20].

**Figure 3:**
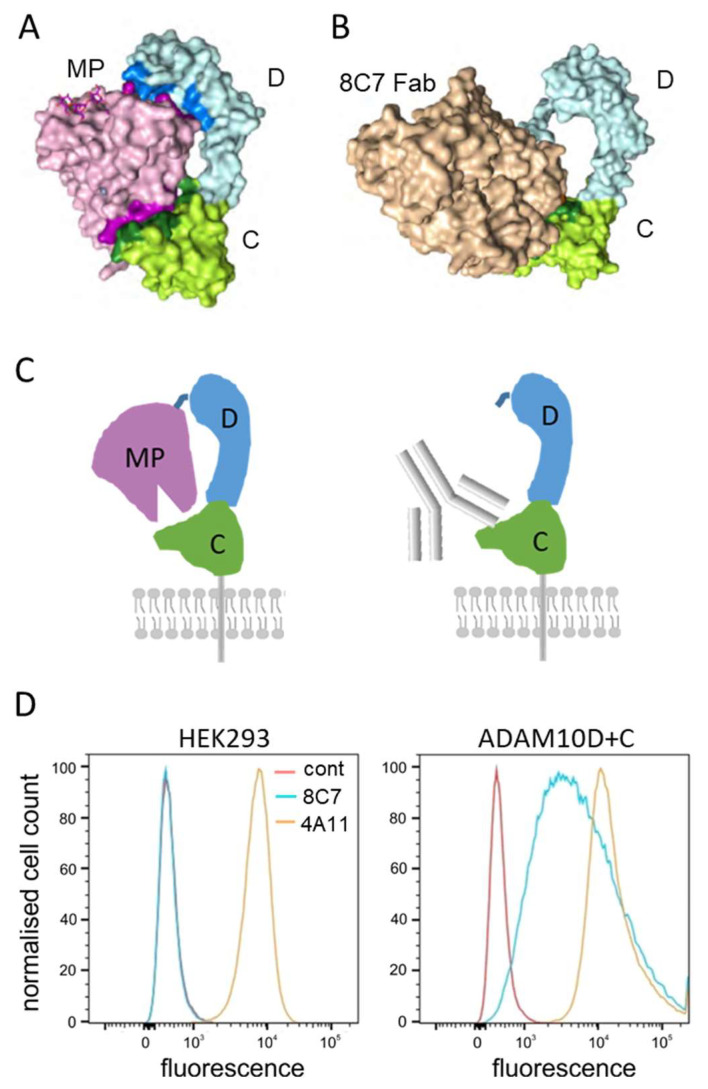
Binding of 8C7 to ADAM10 is sterically regulated by the metalloprotease domain obstructing the 8C7 epitope. (**A**) Crystal structure of the ADAM10 metalloprotease (MP), disintegrin (D) and cysteine-rich (C) domains [13]. (**B**) Crystal structure of the ADAM10 D and C domains in complex with the 8C7 Fab fragment [20]. (**C**) Cartoons of the structures in A and B, illustrating our approach to test whether removal of the MP domain allows binding of 8C7 to ADAM10, using ADAM10 D and C domains lacking the MP domain (ADAM10D + C), expressed on the cell surface. (**D**) HEK293 cells expressing endogenous ADAM10 were transfected with ADAM10D + C and compared to untransfected cells by flow cytometry for binding to the anti-ADAM10 mAbs 8C7 (blue) and 4A11 (yellow), or unstained cells (control, red). (**A**) and (**B**) were adapted with permission from Seegar et al., 2017, Cell [13].

The crystal structure of the ADAM10 extracellular domain suggests a bent, ‘closed’ conformation, where the MP and C domains are in contact, partially obscuring both the catalytic cleft and the C domain [13] (Figure 3A,C). Comparison with the previously determined crystal structure of 8C7 in complex with the isolated D + C domains of ADAM10 (18) showed a steric clash between the MP domain in the closed conformation and 8C7 binding [13] (Figure 3B,C). In addition, 8C7 binding correlates with ADAM activity [13,20], indicating that the selectivity of 8C7 for active ADAM10 may be due to the MP domain inhibiting 8C7 binding in the inactive, closed conformation. To test this hypothesis, we generated a membrane-expressed ADAM10 construct lacking the MP domain (ADAM10D + C), which we transfected into HEK293 cells, which also express endogenous wild type (wt) ADAM10. Flow cytometry showed that 8C7 clearly bound to cells expressing ADAM10D + C on the cell surface, but not parental cells with only the wtADAM10, whereas 4A11 was able to bind to both forms (Figure 3D). This data indicates that 8C7 is selective for an ADAM10 conformation where the C domain is exposed via displacement of the MP domain. ADAM10D + C-expressing cells thus provide a convenient model for testing 8C7 in vitro as a potential means to target cytotoxic payloads in cells with active, ‘open’ ADAM10.

**Figure 4:**
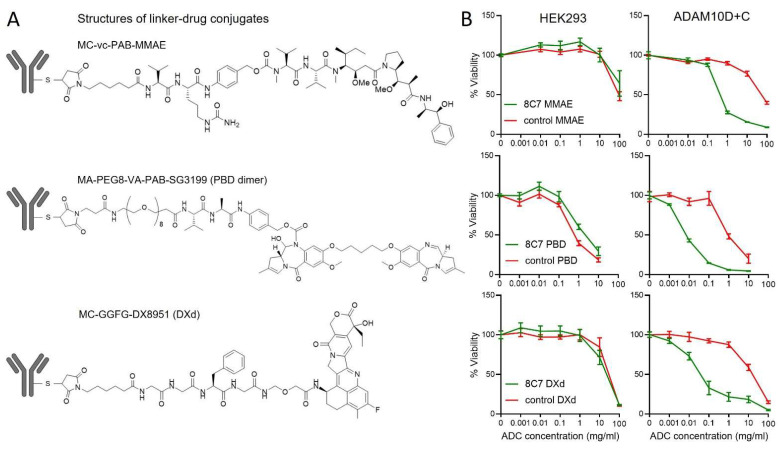
8C7-ADCs specifically kill cells expressing ADAM10 with the exposed C domain epitope. (**A**) Structures of drugs (MMAE, PBD or DXd) and linkers conjugated to 8C7 and control IgG. (**B**) Cell viability of HEK293 cells expressing an ADAM10 construct lacking the metalloprotease domain (ADAM10D + C), or parental HEK293 cells, after treatment with the indicated doses of 8C7 ADCs or matched control IgG ADCs. Cell viability was tested after 3 days by MTS assay. Graphs show mean +/− SEM (*n* ≥ 4).

Next, we generated 8C7 antibody–drug conjugates, by coupling 8C7 with the DNA binding cytotoxin PBD, the microtubule disruptor MMAE, or the Topoisomerase I inhibitor DXd (DX8591 derivative), all of which have been clinically tested with other antibodies [24,25]. The drugs were attached using a cleavable linker, enabling their release upon internalisation into cells. Linker-ADC structures are shown in Figure 4A. Flow cytometry analysis confirmed 8C7-ADCs retained binding to ADAM10D + C cells (Figure S2). We then tested the effects of the ADCs on the viability of ADAM10D + C cells and parental HEK293 cells expressing only endogenous ADAM10. All 8C7-ADCs were able to efficiently kill cells expressing ADAM10D + C containing the exposed 8C7 epitope, compared to the corresponding control ADCs (Figure 4B). In contrast, the parental cells were equally resistant to the control and 8C7-ADCs, confirming the selectivity of 8C7-ADCs. 8C7-PBD had the highest activity, consistent with the known potency characteristics of PBD dimers [24]. Similar to parental HEK293 cells, 8C7-ADCs were ineffective against LIM1215 colon and U251 glioblastoma (GBM) tumour cells expressing ADAM10 (Figure S3), consistent with the low binding of 8C7 to live cells in vitro [19] compared to that in tumours, likely reflecting a predominantly inactive conformation on cells cultured in vitro [20].

**Figure 5:**
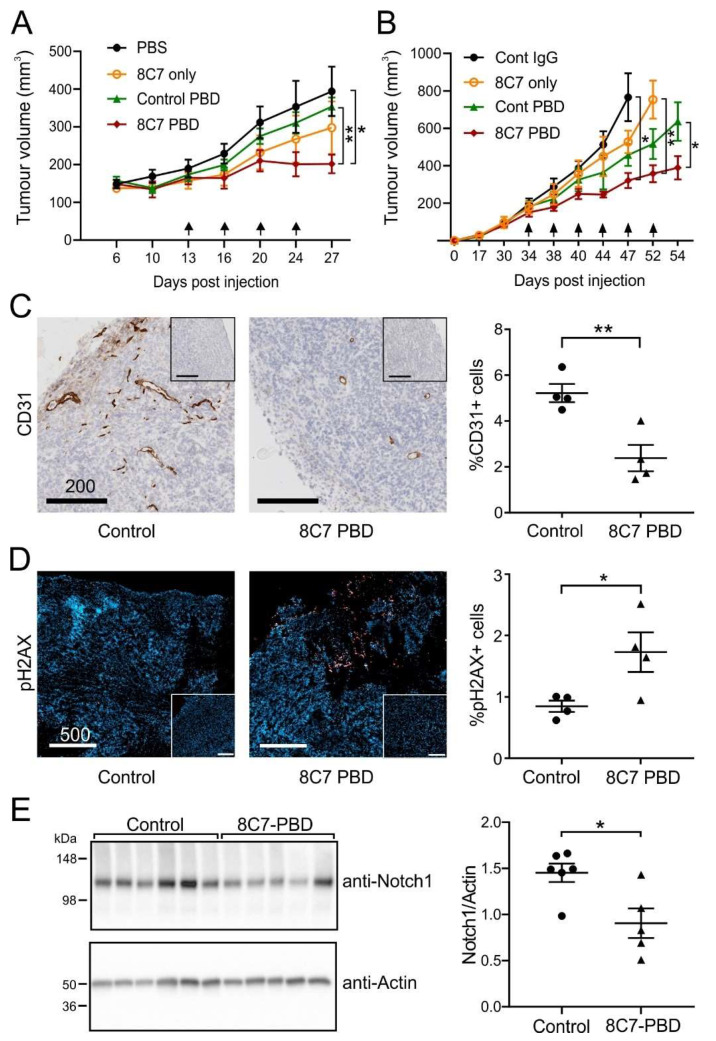
Treatment with 8C7-PBD inhibits tumour growth in mice. (**A**) Tumour volumes in mice bearing LIM1215 xenografts (*n* = 6) treated with 8C7-PBD (0.5 mg/kg), control IgG-PBD (0.5 mg/kg), 8C7 only (10 mg/kg) or vehicle control (PBS) at the indicated times (arrows). (**B**) Mice bearing U251 glioma xenografts (*n* = 5) were treated with 8C7-PBD, control IgG-PBD, control IgG (0.3 mg/kg) or 8C7 only (10 mg/kg) and tumour growth was measured. (**C**,**D**) LIM1215 tumours recovered from control- and 8C7-PBD-treated mice, and analysed via IHC staining of CD31 (**C**, brown) and immunofluorescence staining of phospho-Histone H2AX (**D**, red; nuclei, blue). Insets: secondary antibody-only controls. Representative zoomed images and quantitation of whole tumour sections are shown (scale bars in microns). (**E**) Tumour lysates were Western blotted for Notch 1, and actin was used as loading control. Graph shows quantitation of Notch1 expression relative to actin. All graphs show mean +/− SEM, * *p* < 0.05, ** *p* < 0.01.

Finally, we evaluated 8C7-ADCs in mice bearing tumours. We concentrated on 8C7-PBD, which showed the highest potency in vitro. In addition to LIM1215 colon xenografts, we also used U251 glioblastoma (GBM) xenografts, since ADAM10 expression is associated with poor prognosis in GBM [26], where it mediates Notch and other oncogenic signalling [27,28]. Mice treated with 8C7-PBD exhibited significantly inhibited tumour growth compared to those treated with vehicle or control IgG, and also compared to mice subjected to treatment with a control IgG-PBD conjugate (Figure 5A,B). 8C7-PBD was also more effective than naked 8C7 at >20-fold higher dose (*p* < 0.01 in GBM model, Figure 5B). Tumours from 8C7-PBD-treated mice showed decreased staining of the endothelial marker CD31, and increased staining for phospho-histone γH2AX, a marker for G2-M cell cycle arrest (Figure 5C,D, Figure S4A), known to result from PBD-induced DNA cross-linking [29]. These results are consistent with 8C7-PBD targeting a subpopulation of tumour cells with high Notch activity, that support angiogenesis [20,30]. Indeed, Western blot analysis of tumour lysates indicated decreased Notch 1 levels in 8C7-PBD-treated tumours, with the one outlier being the largest, most resistant tumour (Figure 5E). Mouse weights and overall health were not significantly affected, and were equivalent to control ADC, consistent with minimal binding of 8C7 to ADAM10 in normal tissues (Figure S4B). 8C7-PBD was also effective against a human colon cancer PDX model (Figure S5A). We also tested 8C7-DXd in mice bearing U251 tumours; this also effectively inhibited tumour growth while being well tolerated, with mouse weights being unaffected compared to control mice (Figure S5B,C).

## 4. Discussion

The metalloprotease ADAM10 is highly expressed in a range of tumour types, where it mediates activation of signalling pathways associated with tumour ‘stemness’, proliferation and angiogenesis. Accordingly, its higher expression in tumours correlates with poor patient prognosis for a range of tumour types, suggesting ADAM10 is a potential therapeutic target [3]. Previous attempts to target ADAM10 have focused on small molecule inhibitors targeting the metalloprotease active site, based on hydroxamate analogues originally used for MMPs. While the efficacy and specificity of such inhibitors have improved, none have yet been approved for therapy [3,31,32].

**Figure 6:**
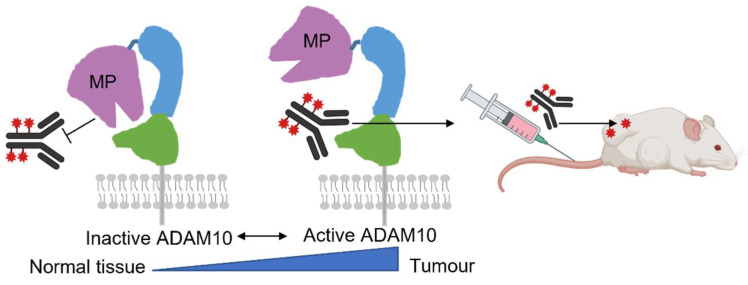
Specificity of mAb 8C7 for an active conformer of the metalloprotease ADAM10 lies in its binding to an epitope in the C domain (green), which is obscured by the metalloprotease domain (MP) in the inactive conformer. Furthermore, 8C7 selectively targets ADAM10 in tumours, and shows improved anti-tumour activity when conjugated to cytotoxic payloads (red).

An alternative approach is to employ monoclonal antibodies, which have the advantages of high affinity, specificity and stability in vivo [33]. They can either be inhibitory (e.g., by competing with ligand or substrate binding), or induce cell death (by immune-mediated cell- or complement-dependent cytotoxicity, or by direct attachment of cytotoxic drugs) [34]. Antibody–drug conjugates (ADCs) are prominent amongst newly approved cancer treatments [35,36]. Clearly, these must preferentially target tumour antigens to avoid toxicity to normal tissues. While the wide expression of ADAM10 in normal tissues could undermine such an approach [37], we previously showed preferential binding of our antibody, 8C7, to active ADAM10 which was upregulated in tumours compared to normal tissues, both in mice and in human samples [20]. We now support this with data from biodistribution studies of radiolabelled 8C7 in mice bearing human colorectal cancer xenografts, in which the mouse/human cross-reactive antibody preferentially accumulated in tumours compared to normal tissues. Furthermore, we show that the activity-dependent selectivity of 8C7 is likely due to conformational exposure of the 8C7 epitope in the C-domain of active ADAM10, which is obscured by the MP domain in the auto-inhibited conformation [13], since removal of the MP domain facilitates robust binding of 8C7. While previous studies have indicated conformational changes upon activation of ADAM proteases [14,15,16], and the ADAM10 crystal structure shows a ‘closed’ inactive conformation, with interaction of the MP and C domains likely causing steric hindrance of substrate binding [13], this is the first direct evidence that movement of the metalloprotease domain allows access to a hidden epitope in the C domain recognized by 8C7. The selectivity of 8C7 for active ADAM10 in tumours [20] is consistent with active ADAM10 having an open structure, allowing substrate and antibody access (Figure 6).

We previously showed partial inhibition of tumour growth in mice using naked 8C7 alone, likely due to it inhibiting ADAM10-mediated Notch activation [20]. Based on its demonstrated tumour selectivity, we sought to improve its anti-tumour efficacy by developing 8C7-ADCs coupled with cytotoxic drugs to inhibit cell replication. We tested 8C7 coupled to a microtubule inhibitor, MMAE, a DNA cross-linker, PBD dimer SGN3199, and a Topoisomerase I inhibitor (DXd). 8C7-ADCs inhibited cell proliferation and viability in vitro compared to matched control IgG-ADCs, with selectivity for cells expressing ADAM10 with the 8C7 epitope exposed. Importantly, experiments in mice confirmed 8C7-ADCs can inhibit growth of colorectal and glioblastoma xenografts, compared to control IgG-ADCs, or 8C7 alone. The effects on tumour growth indicated 8C7-ADCs targeted a subpopulation of tumour cells which exhibited cell cycle arrest, and this was associated with decreased blood vessel density and Notch1 expression, consistent with previous work showing selectivity for tumour stem-like cells with high Notch activity [20]. 8C7-ADCs also appeared well-tolerated at the concentrations used in our experiments, with only a slight trend of weight loss in mice treated with PBD ADCs (both 8C7 and control IgG), indicating minimal non-antigen-related uptake in normal tissues. However, since other studies in animals and humans have shown toxicity of ADCs using PBD dimers [38], we also tested 8C7 coupled to the new generation topoisomerase I inhibitor DXd, used in the anti-HER2 ADC Trastuzumab deruxtecan (DS-8201a, or T-DXd) [25]. 8C7-DXd also effectively inhibited the growth of U251 GBM xenografts in mice with no apparent adverse effects.

## 5. Conclusions

In summary, we show evidence for conformation-specific binding of antibody 8C7 to ADAM10, and its tumour selectivity in mice, supporting development of 8C7-based ADCs. We furthermore show promising activity of our novel 8C7-ADCs in colorectal and glioblastoma xenograft models. This is the first demonstration of the use of ADCs against ADAM proteases as a basis for tumour-selective targeting. Our results indicate a potential for clinical development of ADCs utilising humanised 8C7, which we are currently progressing.

## Data Availability

The data presented in this study are available in this article and the Supplementary Materials.

## Supplementary Materials

The following supporting information can be downloaded at: https://www.mdpi.com/article/10.3390/cancers14133171/s1, Figure S1. Quantitative reverse transcriptase PCR of ADAM10 mRNA from human colorectal tumours compared to nearby normal tissue. Figure S2. Flow cytometry analysis comparing 8C7 and 8C7-ADC binding to HEK293 cells with the exposed 8C7 epitope (ADAM10D + C). Figure S3. Viability assays of tumour cells treated with 8C7-ADCs and matched control ADCs. Figure S4. A. Analysis of U251 tumours from 8C7-PBD-treated mice, and B. Monitoring safety of PBD ADCs. Figure S5. A. Tumour volumes following 8C7-PBD treatment of NSG mice bearing human colon cancer patient-derived xenografts. B, C. 8C7-DXd treatment of NSG mice bearing U251 cell xenografts.

## References

[1] Murphy G. (2008). The ADAMs: Signalling scissors in the tumour microenvironment. Nat. Rev. Cancer.

[2] Atapattu L., Lackmann M., Janes P.W. (2014). The role of proteases in regulating Eph/ephrin signalling. Cell Adhes. Migr..

[3] Smith T.M., Tharakan A., Martin R.K. (2020). Targeting ADAM10 in Cancer and Autoimmunity. Front. Immunol..

[4] Hartmann D., de Strooper B., Serneels L., Craessaerts K., Herreman A., Annaert W., Umans L., Lubke T., Lena Illert A., von Figura K. (2002). The disintegrin/metalloprotease ADAM 10 is essential for Notch signalling but not for alpha-secretase activity in fibroblasts. Hum. Mol. Genet..

[5] Bozkulak E.C., Weinmaster G. (2009). Selective Use of ADAM10 and ADAM17 in Activation of Notch1 Signaling. Mol. Cell. Biol..

[6] Ranganathan P., Weaver K.L., Capobianco A.J. (2011). Notch Signalling in Solid Tumours: A Little Bit of Everything But not all the Time. Nat. Rev. Cancer.

[7] Schumacher N., Rose-John S., Schmidt-Arras D. (2020). ADAM-Mediated Signalling Pathways in Gastrointestinal Cancer Formation. Int. J. Mol. Sci..

[8] Matthews A.L., Szyroka J., Collier R., Noy P.J., Tomlinson M.G. (2017). Scissor sisters: Regulation of ADAM10 by the TspanC8 tetraspanins. Biochem. Soc. Trans..

[9] Tosetti F., Alessio M., Poggi A., Zocchi M.R. (2021). ADAM10 Site-Dependent Biology: Keeping Control of a Pervasive Protease. Int. J. Mol. Sci..

[10] Seegar T.C., Blacklow S.C. (2019). Domain integration of ADAM family proteins: Emerging themes from structural studies. Exp. Biol. Med..

[11] Janes P.W., Saha N., Barton W.A., Kolev M.V., Wimmer-Kleikamp S.H., Nievergall E., Blobel C.P., Himanen J.P., Lackmann M., Nikolov D.B. (2005). Adam Meets Eph: An ADAM Substrate Recognition Module Acts as a Molecular Switch for Ephrin Cleavage In trans. Cell.

[12] Le Gall S.M., Maretzky T., Issuree P.D., Niu X.D., Reiss K., Saftig P., Khokha R., Lundell D., Blobel C.P. (2010). ADAM17 is regulated by a rapid and reversible mechanism that controls access to its catalytic site. J. Cell Sci..

[13] Seegar T.C.M., Killingsworth L.B., Saha N., Meyer P.A., Patra D., Zimmerman B., Janes P.W., Rubinstein E., Nikolov D.B., Skiniotis G. (2017). Structural Basis for Regulated Proteolysis by the alpha-Secretase ADAM10. Cell.

[14] Wang Y., Herrera A.H., Li Y., Belani K.K., Walcheck B. (2009). Regulation of Mature ADAM17 by Redox Agents for L-Selectin Shedding. J. Immunol..

[15] Willems S.H., Tape C.J., Stanley P.L., Taylor N.A., Mills I.G., Neal D.E., McCafferty J., Murphy G. (2010). Thiol isomerases negatively regulate the cellular shedding activity of ADAM17. Biochem. J..

[16] Dusterhoft S., Jung S., Hung C.W., Tholey A., Sonnichsen F.D., Grotzinger J., Lorenzen I. (2013). Membrane-proximal domain of a disintegrin and metalloprotease-17 represents the putative molecular switch of its shedding activity operated by protein-disulfide isomerase. J. Am. Chem. Soc..

[17] Krossa S., Scheidig A.J., Grötzinger J., Lorenzen I. (2018). Redundancy of protein disulfide isomerases in the catalysis of the inactivating disulfide switch in A Disintegrin and Metalloprotease 17. Sci. Rep..

[18] Fischer O.M., Hart S., Gschwind A., Prenzel N., Ullrich A. (2004). Oxidative and Osmotic Stress Signaling in Tumor Cells Is Mediated by ADAM Proteases and Heparin-Binding Epidermal Growth Factor. Mol. Cell. Biol..

[19] Atapattu L., Saha N., Llerena C., Vail M.E., Scott A.M., Nikolov D.B., Lackmann M., Janes P.W. (2012). Antibodies binding the ADAM10 substrate recognition domain inhibit Eph function. J. Cell Sci..

[20] Atapattu L., Saha N., Chheang C., Eissman M.F., Xu K., Vail M.E., Hii L., Llerena C., Liu Z., Horvay K. (2016). An activated form of ADAM10 is tumor selective and regulates cancer stem-like cells and tumor growth. J. Exp. Med..

[21] Sun M.M.C., Beam K.S., Cerveny C.G., Hamblett K.J., Blackmore R.S., Torgov M.Y., Handley F.G.M., Ihle N.C., Senter P.D., Alley S.C. (2005). Reduction-alkylation strategies for the modification of specific monoclonal antibody disulfides. Biocon. Chem..

[22] Vearing C., Lee F.T., Wimmer-Kleikamp S., Spirkoska V., To C., Stylianou C., Spanevello M., Brechbiel M., Boyd A.W., Scott A.M. (2005). Concurrent binding of anti-EphA3 antibody and ephrin-A5 amplifies EphA3 signaling and downstream responses: Potential as EphA3-specific tumor-targeting reagents. Cancer Res..

[23] Fujita K., Chakarov S., Kobayashi T., Sakamoto K., Voisin B., Duan K., Nakagawa T., Horiuchi K., Amagai M., Ginhoux F. (2019). Cell-autonomous FLT3L shedding via ADAM10 mediates conventional dendritic cell development in mouse spleen. Proc. Natl. Acad. Sci. USA.

[24] Beck A., Goetsch L., Dumontet C., Corvaia N. (2017). Strategies and challenges for the next generation of antibody-drug conjugates. Nat. Rev. Drug Discov..

[25] Ogitani Y., Aida T., Hagihara K., Yamaguchi J., Ishii C., Harada N., Soma M., Okamoto H., Oitate M., Arakawa S. (2016). DS-8201a, A Novel HER2-Targeting ADC with a Novel DNA Topoisomerase I Inhibitor, Demonstrates a Promising Antitumor Efficacy with Differentiation from T-DM1. Clin. Cancer Res..

[26] Qu M., Qiu B.O., Xiong W., Chen D., Wu A. (2015). Expression of a-disintegrin and metalloproteinase 10 correlates with grade of malignancy in human glioma. Oncol. Lett..

[27] Nandhu M.S., Hu B., Cole S.E., Erdreich-Epstein A., Rodriguez-Gil D.J., Viapiano M.S. (2014). Novel paracrine modulation of Notch-DLL4 signaling by fibulin-3 promotes angiogenesis in high-grade gliomas. Cancer Res..

[28] Venkatesh H.S., Tam L.T., Woo P.J., Lennon J., Nagaraja S., Gillespie S.M., Ni J., Duveau D.Y., Morris P.J., Zhao J.J. (2017). Targeting neuronal activity-regulated neuroligin-3 dependency in high-grade glioma. Nature.

[29] Flynn M.J., Zammarchi F., Tyrer P.C., Akarca A.U., Janghra N., Britten C.E., Havenith C.E., Levy J.N., Tiberghien A., Masterson L.A. (2016). ADCT-301, a Pyrrolobenzodiazepine (PBD) Dimer-Containing Antibody-Drug Conjugate (ADC) Targeting CD25-Expressing Hematological Malignancies. Mol. Cancer Ther..

[30] Lu J., Ye X., Fan F., Xia L., Bhattacharya R., Bellister S., Tozzi F., Sceusi E., Zhou Y., Tachibana I. (2013). Endothelial Cells Promote the Colorectal Cancer Stem Cell Phenotype through a Soluble Form of Jagged-1. Cancer Cell.

[31] Malemud C.J. (2019). Inhibition of MMPs and ADAM/ADAMTS. Biochem. Pharmacol..

[32] Saha N., Robev D., Himanen J.P., Nikolov D.B. (2019). ADAM proteases: Emerging role and targeting of the non-catalytic domains. Cancer Lett..

[33] Scott A.M., Wolchok J.D., Old L.J. (2012). Antibody therapy of cancer. Nat. Rev. Cancer.

[34] Janes P.W., Vail M.E., Gan H.K., Scott A.M. (2020). Antibody Targeting of Eph Receptors in Cancer. Pharmaceuticals.

[35] Khongorzul P., Ling C.J., Khan F.U., Ihsan A.U., Zhang J. (2020). Antibody-Drug Conjugates: A Comprehensive Review. Mol. Cancer Res..

[36] Hafeez U., Parakh S., Gan H.K., Scott A.M. (2020). Antibody-Drug Conjugates for Cancer Therapy. Molecules.

[37] Wetzel S., Seipold L., Saftig P. (2017). The metalloproteinase ADAM10: A useful therapeutic target?. Biochim. Biophys. Acta.

[38] Saber H., Simpson N., Ricks T.K., Leighton J.K. (2019). An FDA oncology analysis of toxicities associated with PBD-containing antibody-drug conjugates. Regul. Toxicol. Pharmacol..

