# Supplementary material for: Preferential Antibody and Drug Conjugate Targeting of the ADAM10 Metalloprotease in Tumours
Source: Cancers (Basel). 2022 Jun 28;14(13):3171. doi: 10.3390/cancers14133171 (PMC9264901; doi:10.3390/cancers14133171)
Supplement: Supplementary file 1 [file cancers-14-03171-s001.zip › cancers-1731280-supplementary.pdf]

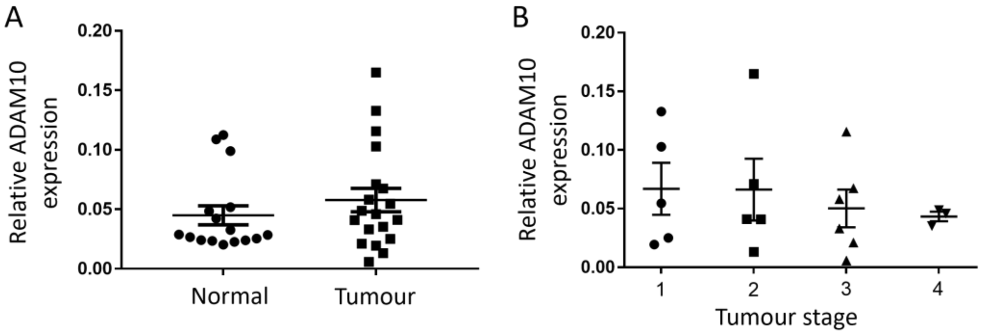

Figure S1. Quantitative reverse transcriptase PCR of ADAM10 mRNA from human colorectal tumours compared to nearby normal tissue (**A**), and by tumour stage (**B**), relative to housekeeping gene expression. Graph shows mean and standard error.

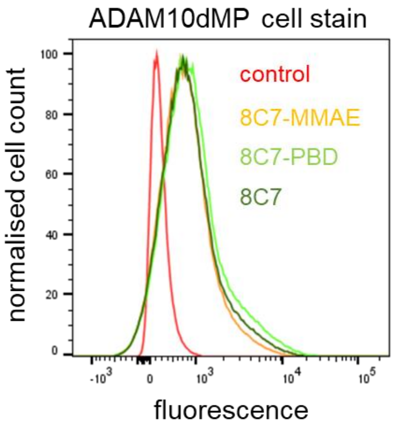

Figure S2. Flow cytometry analysis comparing 8C7 and 8C7-ADC binding to HEK293 cells expressing ADAM10 with the exposed 8C7 epitope (ADAM10D+C).

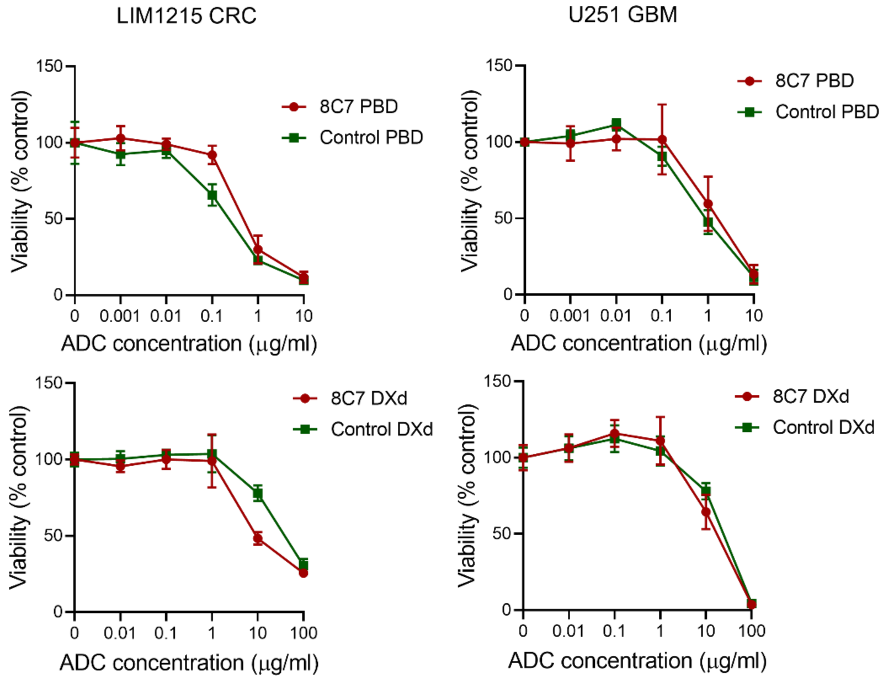

Figure S3. Viability assays of tumour cells (LIM1215 colon and U251 Glioblastoma) treated with 8C7-ADCs and matched control ADCs.

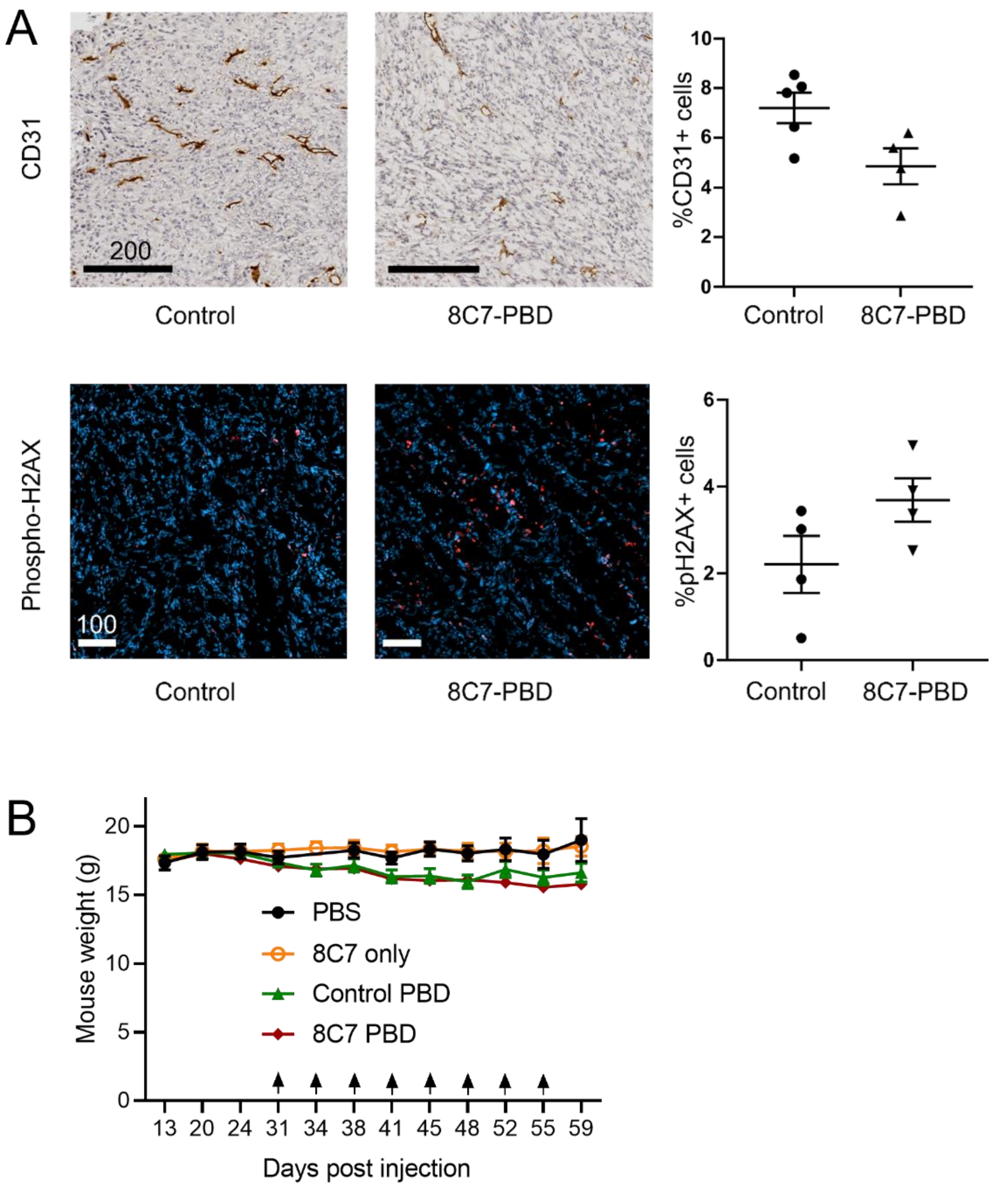

Figure S4. A. Decreased CD31 staining of U251 GBM tumours from 8C7-PBD-treated mice. Tumours from control and 8C7-PBD treated mice (Figure 5A) were recovered and analysed for CD31 expression by IHC (top) or pHistone H2AX staining by IF (bottom). Graphs show quantitation of staining of whole tumour sections. **B.** Monitoring safety of PBD ADCs. Weight of mice (BALB/c nude) treated twice-weekly (arrows) with 8C7-PBD (0.5mg/kg), control IgG-PBD (0.5mg/kg), naked 8C7 (10 mg/kg) or vehicle control (PBS), during 4 weeks of treatment.

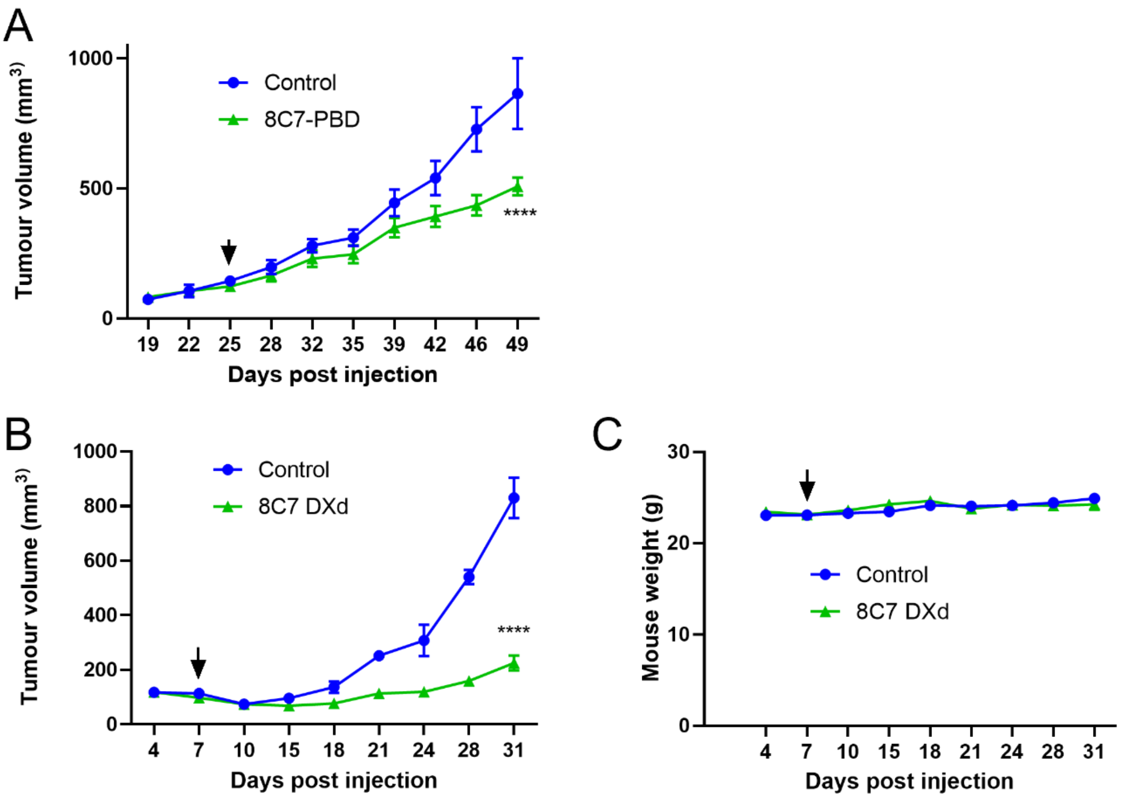

Figure S5. A. Tumour volumes of NSG mice bearing human colon cancer patient-derived xenografts treated with 8C7-PBD, versus vehicle control (in house PDX line 17CH013). Treatment was twice-weekly at 0.5 mg/kg (from arrow). B, C. 8C7-DXd treatment of NSG mice bearing U251 cell xenografts. Treatment was twice-weekly at 4 mg/kg (from arrow), versus vehicle control. Graphs show mean tumour volumes (**B**), and mouse weights (**C**), with standard error bars.

Supplementary methods - Preparation of ADCs

mAb-MC-vc-PAB-MMAE and mAb-MA-PEG8-va-PAB-SG3199 ADCs were prepared according to methods adapted from Sun, et al. (Bioconjug Chem. 2005; 16(5): 1282–1290) for maleimide drug-linker conjugation to antibody inter-chain cysteine residues following partial reduction with tris(2-carboxyethyl)phosphine) (TCEP). Briefly, TCEP in 2.75-fold molar excess was added to antibody that had been previously buffer exchanged into pH 7 phosphate buffer containing EDTA. After incubation at 37°C for 2h, MC-vc-PAB-MMAE or MA-PEG8-va-PAB-SG3199 dissolved in DMSO was added in 6-fold molar excess to the antibody solution. After thorough mixing and incubation at ambient temperature for 2h, the crude conjugation reaction was analyzed by HIC-HPLC chromatography to confirm reaction completion (disappearance of starting antibody peak) at 280 nm wavelength detection. Purification of the resulting ADC was then carried out by gel-filtration chromatography using an AKTA Pure system equipped with a Sephadex desalting column (Cytiva) equilibrated with PBS. The average drug-to-antibody ratio (DAR) was calculated to be 3.7 – 4.2 based on comparative peak area integration of the HIC-HPLC chromatogram along with UV-Vis analysis utilizing the molar extinction coefficients of the antibody and drug-linker. Confirmation of low percent high molecular weight (HMW) aggregates for the resulting ADC was determined using analytical SEC-HPLC. Final ADC products were then sterile filtered, flash frozen, and stored at -80°C until further evaluation.

mAb-MC-GGFG-Dx8951 ADC was prepared, purified, and characterized as above, but with the following modifications to the conjugation protocol: 12-fold molar excess of TCEP was added to the starting antibody mixture, followed by buffer exchange into fresh phosphate/EDTA buffer immediately prior to final addition of 12-fold molar excess of MC-GGFG-Dx8951.
